# Emergent Radiotherapy for Leukemia-Induced Cranial Neuropathies Refractory to Intrathecal Therapy

**DOI:** 10.7759/cureus.15212

**Published:** 2021-05-24

**Authors:** Nirav Patel, Benjamin J RIch, Shareen Patel, Justin M Watts, Ronald Benveniste, Matthew Abramowitz, Arnold Markoe, Daniel G Eichberg, Ricardo J Komotar, Marcarena De La Fuente, Joshua Pasol, Tejan Diwanji

**Affiliations:** 1 Radiation Oncology, University of Miami Sylvester Comprehensive Cancer Center, Miami, USA; 2 Radiation Oncology, University of Miami Miller School of Medicine, Miami, USA; 3 Hematology/Oncology, University of Miami Sylvester Comprehensive Cancer Center, Miami, USA; 4 Neurological Surgery, University of Miami Miller School of Medicine, Miami, USA; 5 Neuro-Oncology, University of Miami Sylvester Comprehensive Cancer Center, Miami, USA; 6 Ophthalmology, University of Miami Sylvester Comprehensive Cancer Center, Miami, USA

**Keywords:** cns manifestations, palliative radiation therapy, rare eye disorders, whole brain radiation, acute myeloid leukemia (aml)

## Abstract

Neurologic symptoms from leukemic infiltration of the central nervous system are an oncologic emergency, and expeditious treatment is required to preserve function. We report the case of a 44-year-old patient with relapsed acute myeloid leukemia (AML) who developed sub-acute cranial neuropathies refractory to treatment with intrathecal (IT) chemotherapy. The patient was therefore treated with an emergent course of whole-brain radiotherapy, resulting in immediate improvement and subsequent resolution of cranial neuropathies. This case illustrates that while central nervous system involvement by AML is rare, radiotherapy remains an effective modality to avoid long-term morbidity in patients failing to respond to systemic or IT chemotherapy.

## Introduction

While central nervous system (CNS) involvement of acute myeloid leukemia (AML) is rare, the onset of neurologic symptoms can have catastrophic, irreversible consequences leading to significant morbidity and diminished quality of life [1-3]. Rapid initiation of treatment is crucial to preserve neurologic function, and special consideration must be given when utilizing chemotherapy with radiotherapy (RT) [4-6]. We report the case of a patient with relapsed AML who presented with cranial nerve (CN) palsies unresponsive to intrathecal (IT) cytarabine and methotrexate (MTX), requiring emergent whole-brain radiotherapy (WBRT).

## Case presentation

**Figure 1:**
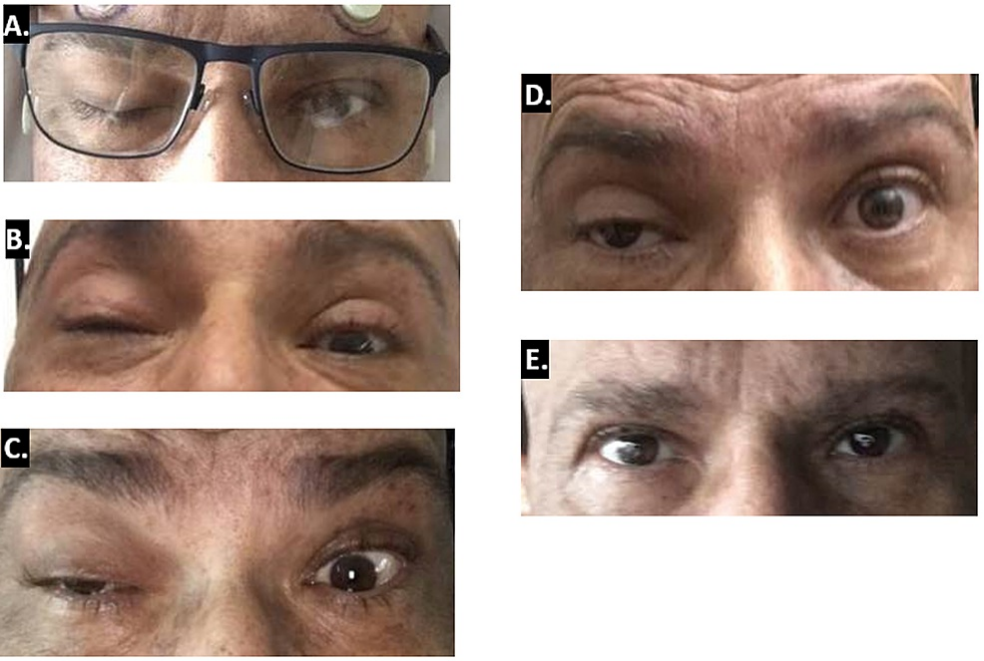
Right eye ptosis at presentation and over the course of radiotherapy. (A) Patient at presentation to the emergency department with right eye ptosis. (B) Patient after two fractions of radiotherapy noted to have mild improvement in his right eye ptosis. (C) Patient at completion of five fractions of radiotherapy with continuing improvement in right eye ptosis. (D) Patient two days after completion of radiotherapy noted to have significant improvement in right eye ptosis. (E) Patient at one month following completion of radiotherapy with resolution of right eye ptosis.

**Figure 2:**
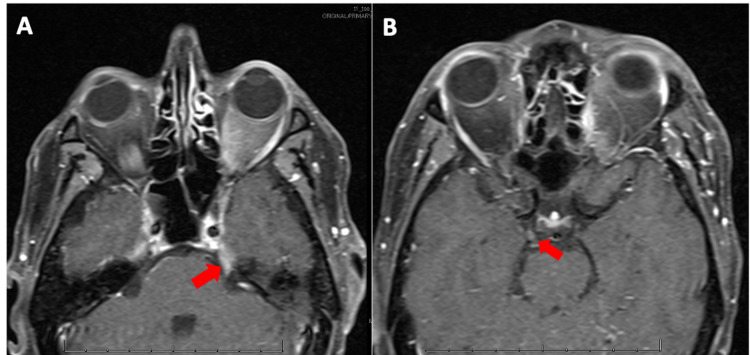
Axial T1 post-contrast MRI images. (A) Red arrow pointing to abnormal enhancement of the cisternal segment of the left trigeminal nerve. (B) Red arrow pointing to abnormal enhancement involving the distal cisternal and cavernous segment of the right oculomotor nerve.

A 44-year-old man was diagnosed with AML with a normal karyotype and FLT3-ITD and WT1 mutations. He was induced with 7+3 regimen (cytarabine and high-dose daunorubicin) and gemtuzumab ozogamicin. Post-remission therapy included two cycles of high-dose cytarabine with midostaurin followed by a myeloablative allogeneic stem cell transplant (conditioning regimen did not include RT). Approximately three months post-transplant, the patient had systemic relapse and was treated with hydroxycarbamide for cytoreduction followed by clofarabine, cytarabine, and sorafenib re-induction, and achieved molecular complete remission. Two months later, however, he presented to the emergency department with three weeks of diplopia and multiple CN palsies. At presentation, examination was notable for right eye ptosis and ophthalmoplegia signifying a right CN III, IV, and VI palsy (Figure 1A). A brain MRI revealed abnormal CN enhancement of the right oculomotor and left trigeminal nerves suggestive of CNS recurrence (Figures 2A, 2B). Cerebrospinal fluid (CSF) analysis by cytology and flow cytometry revealed neoplastic myeloid blasts, confirming recurrent disease. A bone marrow biopsy was negative for systemic involvement.

**Figure 3:**
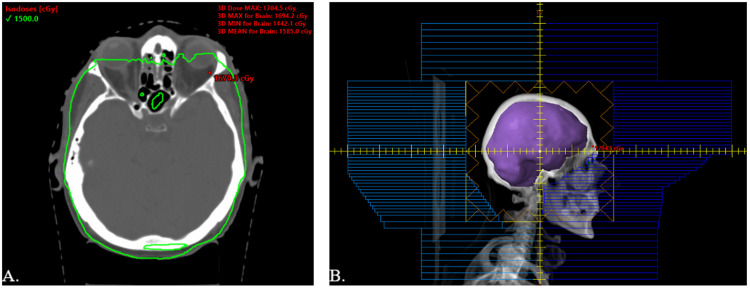
Radiation treatment plan. (A) Radiation treatment plan of 15 Gy in five fractions using 6-MV opposed lateral photon beams. (B) Digitally reconstructed radiograph of the treatment field with an inferior border at C2-C3.

The patient was started on IT MTX 12 mg, cytarabine 50 mg, and hydrocortisone 50 mg, with daily gilteritinib 120 mg per orem. He received two cycles of IT MTX, cytarabine, and hydrocortisone without symptomatic improvement, at which time the decision was made to proceed with RT. RT was initiated five days after his last IT MTX and three days after his last IT cytarabine to allow for washout. He received a dose of 15 Gy in five fractions using three-dimensional conformal WBRT via 6-MV opposed lateral photon beams (Figures 3A, 3B). This radiation dose was chosen to reduce the risk of toxicity with concurrent MTX and permit the safe receipt of consolidative craniospinal irradiation should it be needed. A dose of 3 Gy per fraction was chosen because of the malignancy’s resistance to prior chemotherapy. After two fractions of RT, there was an improvement in his right eye ptosis (Figure 1B). At the completion of RT, his CN palsies continued to improve (Figures 1C, 1D), and he received an additional cycle of IT MTX, cytarabine, and hydrocortisone. One month following completion of RT, his CSF was negative for blasts, and he had complete resolution of his right CN palsies and diplopia (Figure 1E). High-dose MTX was not administered prior to, or after, his course of RT. Six months following RT, he continued to do well on maintenance gilteritinib without neurologic or systemic relapse. He did not have significant or long-term toxicities from RT.

## Discussion

The age-adjusted incidence of AML in the United States is approximately 4.3 per 100,000 persons, with a median age at diagnosis of 68 years [7]. CNS involvement occurs in approximately 3% of AML patients [8]. IT chemotherapy is the initial treatment of choice in AML patients with CNS involvement due to its ability to rapidly clear the CSF. Overall, the use of RT for CNS leukemia has reduced with time due to toxicity concerns; however, it continues to play an important role in cases where there is CN involvement, cord impingement, or failure to respond to IT chemotherapy. The decision to proceed with RT in this patient with CN involvement required special consideration regarding radiation fields, dose/fractionation, and timing of RT given the receipt of IT chemotherapy in the preceding days.

MTX is an antimetabolic drug that interferes with folate metabolism [9]. Leukoencephalopathy is the most devastating complication of MTX, and combination with RT is often avoided due to concerns of synergistic toxicity [10]. The risk of developing leukoencephalopathy is directly related to the use of combination RT, dose of cranial irradiation, dose of MTX, and sequence of administration. The risk is lower when MTX is given prior to RT and maximal when MTX is given concurrently with or soon after RT [11].

Our patient received two cycles of IT MTX prior to radiation oncology consultation, and the optimal timing for initiation of RT was uncertain. IT MTX is eliminated in a biphasic pattern with half-lives of 4.5 hours and 14 hours [12]. In order to allow for adequate washout of MTX and reduce the risk of leukoencephalopathy, the decision was made to delay RT.

In contrast, the half-life of IT cytarabine is shorter than MTX (2-6 hours in the CSF) [13]. One additional cycle of IT cytarabine was therefore delivered during this MTX washout period to avoid progression until initiation of RT.

It was unclear in this patient if the ideal RT treatment field would encompass the base of skull (BOS) alone, whole brain, or entire craniospinal axis. Walker et al. published a retrospective analysis of patients with CNS leukemic involvement in which improved CNS progression-free survival was noted in patients receiving craniospinal axis/WBRT compared to BOS RT (77% vs. 51%; p = 0.02) [5]. Patients with negative bone marrow involvement who received BOS RT had a 12-month CNS progression-free survival of 47% compared to 77% in those treated with a larger field. The majority of patients treated in an analysis from Ha et al. were also treated with WBRT (93%) vs. BOS RT (7%) [14]. In light of the above, the patient was treated with WBRT. Craniospinal RT was not recommended due to its increased toxicity, and instead WBRT was used as a bridge to systemic therapy (gilteritinib) with the option to deliver craniospinal RT in the future for consolidative purposes.

Ha et al. previously assessed the effectiveness of RT in reversing CN palsies due to leukemic involvement [14]. Resolution of symptoms was noted in 14 (50%) patients, improvement in eight (29%) patients, and stable or progressive disease in four (14%) patients. Of note, all patients included in their analysis received concomitant IT or systemic chemotherapy.

Paryani et al. reviewed their experience of children with ALL and non-Hodgkin’s lymphomas who developed CN palsies [1]. Out of 20 patients treated, 16 had objective control of their CNS disease, with complete resolution of CN palsies in 14 patients. In their analysis, seven patients received IT chemotherapy prior to RT and only two had improvement in CN palsies with chemotherapy alone, whereas four out of the remaining five patients had symptomatic improvement after RT. Gray and Wallner reviewed the efficacy of RT in reversing CN palsies in 20 patients treated with WBRT to a median dose of 21 Gy and noted a 95% overall response rate and a 44% complete response rate at three months [15].

Due to small patient cohorts in these studies, a dose-response relationship has not been determined. Our patient was treated with a dose of 15 Gy in five fractions to minimize potential toxicities of concurrent MTX and to allow for consolidative craniospinal RT in the future. A dose of 3 Gy per fraction was used to overcome the malignancy’s prior resistance to chemotherapy. The patient had an excellent response to treatment at this dose with persistent resolution of his CN dysfunctions at his last follow-up six months after RT.

## Conclusions

In conclusion, RT can effectively relieve symptoms of leukemia with CNS involvement. The immediate symptomatic response observed in this patient after two fractions of RT indicates that the treatment effect was primarily a result of RT. In fact, in our patient with no neurologic improvement on IT chemotherapy, RT proved to be an extremely effective and efficient treatment in the setting of rapidly deteriorating neurologic function. This case highlights the role of WBRT in the treatment of symptomatic leukemic CNS involvement.

## References

[1] Paryani SB, Donaldson SS, Amylon MD, Link MP (1983). Cranial nerve involvement in children with leukemia and lymphoma. J Clin Oncol.

[2] Laningham FH, Kun LE, Reddick WE, Ogg RJ, Morris EB, Pui CH (2007). Childhood central nervous system leukemia: historical perspectives, current therapy, and acute neurological sequelae. Neuroradiology.

[3] Shihadeh F, Reed V, Faderl S (2012). Cytogenetic profile of patients with acute myeloid leukemia and central nervous system disease. Cancer.

[4] Castagnola C, Nozza A, Corso A, Bernasconi C (1997). The value of combination therapy in adult acute myeloid leukemia with central nervous system involvement. Haematologica.

[5] Walker GV, Shihadeh F, Kantarjian H (2014). Comprehensive craniospinal radiation for controlling central nervous system leukemia. Int J Radiat Oncol Biol Phys.

[6] Pinnix CC, Yahalom J, Specht L, Dabaja BS (2018). Radiation in central nervous system leukemia: guidelines from the International Lymphoma Radiation Oncology Group. Int J Radiat Oncol Biol Phys.

[7] Shallis RM, Wang R, Davidoff A, Ma X, Zeidan AM (2019). Epidemiology of acute myeloid leukemia: recent progress and enduring challenges. Blood Rev.

[8] Rozovski U, Ohanian M, Ravandi F (2015). Incidence of and risk factors for involvement of the central nervous system in acute myeloid leukemia. Leuk Lymphoma.

[9] Bhojwani D, Sabin ND, Pei D (2014). Methotrexate-induced neurotoxicity and leukoencephalopathy in childhood acute lymphoblastic leukemia. J Clin Oncol.

[10] Verstappen CC, Heimans JJ, Hoekman K, Postma TJ (2003). Neurotoxic complications of chemotherapy in patients with cancer: clinical signs and optimal management. Drugs.

[11] Keime-Guibert F, Napolitano M, Delattre JY (1998). Neurological complications of radiotherapy and chemotherapy. J Neurol.

[12] Bleyer AW (1977). Clinical pharmacology of intrathecal methotrexate. II. An improved dosage regimen derived from age-related pharmacokinetics. Cancer Treat Rep.

[13] Kwong YL, Yeung DY, Chan JC (2009). Intrathecal chemotherapy for hematologic malignancies: drugs and toxicities. Ann Hematol.

[14] Ha CS, Chung WK, Koller CA, Cox JD (1999). Role of radiation therapy to the brain in leukemic patients with cranial nerve palsies in the absence of radiological findings. Leuk Lymphoma.

[15] Gray JR, Wallner KE (1990). Reversal of cranial nerve dysfunction with radiation therapy in adults with lymphoma and leukemia. Int J Radiat Oncol Biol Phys.

